# Publication Ethics: A Case Series with Recommendations According to Committee on Publication Ethics (COPE)

**Published:** 2012

**Authors:** Bibi Seddigheh Fazly Bazzaz, Ramin Sadeghi

**Affiliations:** 1 *Biotechnology Research Centre, School of Pharmacy, Mashhad University of Medical Sciences, Mashhad, Iran*; 2 *Nuclear Medicine Research Centre, Imam Reza Hospital, Faculty of Medicine, Mashhad University of Medical Sciences, Mashhad, Iran*

**Keywords:** Authorship criteria, Copyright transfer, Data fabrication, Plagiarism, Publication ethics

## Abstract

Ethical misconduct is not a new issue in the history of science and literature. However, ethical misconducts in science have grown considerably in the modern era which is due to emphasis on the scientific proliferation in research institutes and gauging scientists according to their publications. In the current case series, several misconducts occurring over the previous years in Mashhad University of Medical Sciences (Mashhad, Iran) either for Journals or Faculty members were gathered and specific recommendations were provided to avoid similar events in the future. All recommendations are according to Committee on Publication Ethics (COPE).

## Introduction

Ethical misconduct is not a new issue in the history of science and literature. As a classical example, in the 11^th^ century *al-Hajvery al-Ghaznawi *the author of *Revelation of the Veiled *(famous treatise on Sufis) claimed that two of his previous works have been the subjects of plagiarism. He asked the readers not to use his words without citing him as the author. However, a century later *Attar* (famous Persian poet) extensively used *Revelation of the Veiled *in his famous book *Biographies of the Saints* without citing *Al-Hajvery *(1) . 

Since then, the legal aspects of ethical misconducts in science have grown considerably which is due to emphasis on the scientific proliferation in research institutes and gauging scientists according to their publications. In the current case series, several misconducts occurring over the previous years in Mashhad University of Medical Sciences, (Mashhad, Iran) either for Journals Faculty members were gathered and specific recommendations were provided to avoid similar events in the future. All recommendations are according to Committee on Publication Ethics (COPE) (2). 

## Disputes in authorship

***Case:*** A postgraduate medical student complained to the vice chancellery of research of her university that she has not been included in the authors list of an article which was prepared using the results of her thesis. The senior researcher involved in her thesis claimed that she has forgotten to include the student as an author. The issue was discussed and the student refused to pursue the case any further.

***Recommendations***

COPE is very straightforward regarding authorship disputes. For adding or omitting an author, a request should be sent to the publishing journal. The journal will ask the permission of all authors and correction would be made only if all authors consent. In the mentioned case, this could have been done too (3, 4). 

***Case:*** Researcher SR included a senior researcher of another department to the authors list of his article. Although the senior researcher was not aware of, he thanked SR upon receiving a copy of the published article in a prestigious journal.

***Recommendations***

This is a clear case of guest or gift authorship. It is not recommended to add a researcher to the authors list of an article if he/she is not involved in the research to justify the authorship. If an editor finds out about a gift authorship, removal of the suspected gift author is the recommendation of COPE. Long authors list (for example for simple case reports) can alert the editors of possible gift authorship (5). It is strongly recommended to state what the authors contributed to the research. Recently, many journals require this form to be completed before manuscript submission. 

## Copyright violation

***Case:*** MK the author of a researcher, aimed to include a figure of a textbook in his manuscript which was going to be submitted to a journal with high impact factor. He asked another co-researcher in this regard. His colleague recommended asking permission to reproduce the figure from the publisher of the book. MK emailed the publisher and permission was granted without any charge.

***Recommendations***

Copyright violation is a common neglected misconduct in countries that do not observe copyright law. Reproducing any part of an article or book (figure, table, etc) definitely needs permission from copyright holder. The copyright holder is usually the publisher not the authors since the authors usually have transferred the copyright to the publisher upon submission of their manuscripts. This is why we should contact publisher not the authors for asking permission. 

**Data fabrication**

***Case:*** A junior researcher published an article in a PUBMED indexed journal. The senior researcher of the organization read the article and noticed the striking resemblance of the article topic with one of his accepted research projects which was still in the patient recruiting phase. They asked the junior researcher for raw data of his research and he was unable to provide the data. Finally he admitted that he fabricated the data to prepare the manuscript. 

***Recommendations***

Data fabrication is a serious act of misconduct which usually goes unnoticed, however, once detected, it can bring disgrace to the researcher, his/her institute or even his/her country. Hwang Woo-suk case of data fabrication is the most famous in this regard (6). Retracting the articles with fabricated data and reporting it to the institutional regulatory body of this misconduct is the recommendation of COPE (7). Universities and research centres should be very sensitive to this important issue by reprimanding or dismissing researchers involved in fabrication. 

**Editorial misconduct**

**Case: **Upon acceptance of a manuscript prepared by researcher (RS), editor in chief of a journal asked him to add an article published in their journal in the reference list of the accepted manuscript. RS accepted and added a reference and the article was published.

***Recommendations***

This is a case of editorial misconduct with the main aim of increasing impact factor. This is discouraged by COPE (8), However, it is widely practiced by the editor in chiefs. The authors should resist this request as much as possible. 

**Local ethics committee and its importance**

***Case:*** HK is a researcher who published an article which was the result of a research project with approval of local ethics committee of his institution. Another researcher expressed his concern regarding the ethical issues of the published article in a letter to editor. The editor in-chief asked HK to provide documents of ethics committee approval. HK provided the documents and explained his point of view in response to the published letter to editor. 

***Recommendations***

This case shows the importance of local ethics committee approval. Journals usually ask authors to clearly mention the approval of ethics committee in their manuscripts. Researchers should get the local ethics committee approval for their research before conducting any experiment on human or animal subjects. Actually COPE recommends its members not to publish any article without appropriate ethics committee approval (9). Editors have the right to contact ethical committees of a researcher to ask further information. It is worth mentioning that, having approval of local ethics committee does not guarantee ethical integrity of a research and editors still can reject researches with local ethics committee approval on the basis of unethical research conduct (10). 

## Multiple submissions

***Case:*** HA submitted a manuscript to two journals simultaneously. The decision of the editorial boards of both journals was acceptance with minor revision. She asked her colleagues for advice. Finally she emailed the editor in-chief of one of the journals and withdrew her submission. The article was published in the other journal.

***Case:*** VD submitted a manuscript to a journal. After couple of months of not hearing from the editorial board, he re-submitted the manuscript to another journal. A day after re-submission, he received an email from the first journal that his article was going to be accepted after minor revisions. He withdrew the re-submission from the second journal and article was published in the first journal. 

***Recommendations***

The authors are usually asked to mention in a signed form that their submission is not under review elsewhere. Any violation of this signed form is considered as misconduct (11). The problem is when editorial board of a journal does not review a manuscript in an appropriate time. The authors can withdraw their manuscript any time they wish due to prolonged unacceptable reviewing process. However, the editor in chief should be informed beforehand and the documents of any correspondence should be kept by the corresponding author. Authors should never submit a manuscript to another journal before getting rejected or appropriate withdrawal of the manuscript from the first journal.

**Redundant publication**

***Case:*** RB is a researcher who had previously published an article in a local journal of his country in his mother tongue. He prepared another article of his research in English and submitted it to another journal. The manuscript was accepted for publication. However, the editor in-chief of the English journal managed to find out about the first article and asked the author to clarify the issue. RB asked for a permission from the first journal to publish the article in English in another journal which was granted. The English article was published and the case was resolved.

***Recommendations***

This is a case of redundant publication. The authors usually are asked to give a signed statement that the manuscript they are submitting has not been published elsewhere. Any violation of this statement is misconduct and can end in retraction of a published article. If a translation of a previously published article is going to be submitted to another journal, permission of the first journal should be taken beforehand with appropriate reference to the first publication (12).

## Plagiarism

***Case:*** A junior researcher prepared a manuscript from the results of a thesis and the manuscript was published in a prestigious journal. A month after publication, the junior researcher was contacted from the editor in-chief with the concern of plagiarism. He responded that he used a ghost writer to prepare the manuscript. The editor in-chief retracted the article due to plagiarism detected in the published article.

***Recommendations***

Plagiarism is a misconduct which is usually acted upon by a junior researchers who are not aware of the legal issues of “copying and pasting” from another article (13). Editors of the journals are checking more and more actively for possible plagiarism and it is recommended to teach the junior researchers the legal aspects of plagiarism. Improving English skills of the researchers can prevent possible future misconducts. 

## Conclusions

Recent surge of published researches and measuring researchers according to their scientific output can implicitly or explicitly cause misconducts in the publication of the research results. All researchers should be aware of these misconducts and their legal consequences. The above mentioned cases indeed happened and can be repeated in the future and some of them can be career ending and very serious. Future misconducts can hopefully be prevented by this case series. 
